# Resilience and limitations of MFC anodic community when exposed to antibacterial agents

**DOI:** 10.1016/j.bioelechem.2020.107500

**Published:** 2020-08

**Authors:** Oluwatosin Obata, John Greenman, Halil Kurt, Kartik Chandran, Ioannis Ieropoulos

**Affiliations:** a Bristol BioEnergy Centre, Bristol Robotics Laboratory, University of the West of England, BS16 1QY, UK; b Biological, Biomedical and Analytical Sciences, University of the West of England, BS16 1QY, UK; c Department of Earth and Environmental Engineering, Columbia University, New York, United States

**Keywords:** Ampicillin, Chloroxylenol, Microbial fuel cell, Anodic biofilm, MFC cascade, Urine

## Abstract

This study evaluates the fate of certain bactericidal agents introduced into microbial fuel cell (MFC) cascades and the response of the microbial community. We tested the response of functioning urine fed MFC cascades using two very different bactericidal agents: a common antibiotic (Ampicillin, 5 g/L) and a disinfectant (Chloroxylenol 4.8 g/L) in concentrations of up to 100 times higher than the usual dose. Results of power generation showed that the established bacteria community was able to withstand high concentrations of ampicillin with good recovery after 24 h of minor decline. However, power generation was adversely affected by the introduction of chloroxylenol, resulting in a 99% loss of power generation. Ampicillin was completely degraded within the MFC cascade (>99.99%), while chloroxylenol remained largely unaffected. Analysis of the microbial community before the addition of the bactericidal agents showed a significant bacterial diversity with at least 35 genera detected within the cascade. Microbial community analysis after ampicillin treatment showed the loss of a small number of bacterial communities and proportional fluctuations of specific strains within the individual MFCs community. On the other hand, there was a significant shift in the bacterial community after chloroxylenol treatment coupled with the loss of at least 13 bacterial genera across the cascade.

## Highlights

• MFC cascade can withstand and degrade high concentrations of ampicillin.
• Chloroxylenol caused loss of power and microbial community within MFC cascade.
• MFC anodic biofilm responds differentially to different inhibitory chemical agents.
• MFC cascade has the potential for bioremediation of certain chemical agents.

## 1 Introduction

Microbial fuel cells (MFCs) are bioelectrical devices convert chemical energy in organic matter directly into electricity as a by-product of anaerobic oxidation of biodegradable organic substrates [1]. Different substrates have been studied for their suitability for power generation in MFCs with varying results. The substrates range from simple low carbon substances e.g. acetate [2] to easily fermentable ones such as glucose and non-readily fermentable complex ones such as those found in wastewater [3] and urine [4].

Recent advances in MFC technology such as novel environmentally friendly materials or optimised designs, among others, have resulted in improved power performance and potential practical applications [4].The increasing numbers of field trials and pilot tests reported in the last few years are indicators of the significant progress made in advancing the real life applications of this technology [5], [6]. This is achieved partly by the adoption of plurality of the MFC units by stacking and cascading [7]. Recently, MFC technology using human urine as the sole substrate have been tested to power lightings in real life settings. An example was the collaboration between the Bristol Bioenergy Centre and the Glastonbury music festival, the largest of such in the UK. The trial consists of twelve MFCs modules arranged in a cascade of 4 modules, electrical energy generated was stored in 8 batteries and used to power 6 LED strips continuously during the event [7]. Other demonstrations and long term performance analysis highlighting the stability of the technology are indication of this continued maturity [8]. Human urine constitutes unique substrate for current generation in microbial fuel cell systems. It contains a wide range of organics and nutrients capable of supporting the growth of certain bacteria with MFCs. Human urine contains high concentration of ammonium ions, ammonia and phosphates as well as creatinine and urea. Another important characteristic of urine is its high conductivity which could enhance the reduction of electrolyte ohmic loses, leading to enhanced electrochemical process [9].

The introduction and implementation of MFC technology in the field (domestic or industrial) means that it would be subject to both natural and anthropogenic elements, which could adversely affect its overall performance. The anodic biofilm of MFCs is composed of complex mix of microbial communities of fermentative and electroactive organisms working together in synergy to achieve efficient degradation of substrates [9], [10]. Nevertheless, the ability of the anodic biofilm to metabolise the feed substrate depends on the nature of the substrate and its constituent concentrations. The anodic biofilm which catalyse the metabolic reactions within the MFC systems are vulnerable to inhibition of contamination. This was brought to light in one of our recent deployments of the technology to a rural community in parts of Africa where different chemical elements were inadvertently introduced into the MFC system.

Ampicillin is a broad spectrum β-lactam based antibiotic used for the treatment of lungs, heart and urinary tract infections. Due to its wide range of its application, ampicillin can be found in different sources including hospital, agricultural and domestic wastewaters. It is however difficult to remove from these sources by conventional wastewater treatment systems due to its photo-resistance [11], [12]. Choloxylenol on the other hand is the active ingredient in some major antiseptics used in homes, such as Dettol. Both of these chemicals are commonly used domestically and as such MFC systems deployed to homes or hospital settings could easily be exposed to them.

There is currently little or no report on the assessments of the impact of inhibitory materials intentionally or accidentally introduced into functioning MFC systems, especially household products. Therefore, it is essential to evaluate the stability of the technology under these conditions, especially the anodic biofilm responsible for power generation. Recent findings have demonstrated the robustness of the developed anodic biofilm towards certain known inhibitory materials as well as its ability to not only withstand some of these inhibitors but also break them down. For instance Zhang, et al. [3] conducted a study which investigated the impact of chloramphenicol- a known antibacterial agent within the anode of a dual-chamber MFC reactors separated by cationic exchange membranes*.* The result of that study showed up to 84% degradation of the antibiotics within 12 h in the MFC. In that study, analysis of the microbial community at the end of the test revealed a substantially diverse community with at least 15 genera detected. *Azonexus* and *Comamonas*, which were the dominant genera within the MFC, are known electroactive species, with the ability to degrade toxic and recalcitrant compounds. The results showed that power generation continued even in the presence of the antibacterial agent [3]. This is an indication that the bacterial community possess sufficient defence mechanisms to mitigate the impact of the agent or was able to directly breakdown the materials into less harmful derivatives.

In another study, the ability of MFC to degrade and transform drugs such as sulphamethoxazole and 3-amino-5-methylisoxazole was evaluated. The result of that study showed that the MFC did not only degrade sulphamethoxazole but also transformed amino-5-methylisoxazole [13]. Furthermore, a removal efficiency of 84.5% was reported for model azo dye methyl orange introduced into a photo-electrocatylitic microbial fuel cell within 35 h. The result of the experiment showed that maximum power generation reached 0.12 W/m^2^ in the presence of the dye [14]. These studies highlight the ability of established MFC biofilms to withstand high concentration of certain inhibitory materials as well as their potential for pollutant degradation while also generating usable electricity.

For a successful development and deployment of MFC technology, the microbial community would need to show high level of tolerance to different inhibitory materials as well as other chemical and biological elements they might be exposed. Recent findings have shown that the developed anodic biofilm was able to withstand and even suppress the presence of large numbers of external pathogenic organisms introduced into the stream [15], [16], which confirms the stability and robustness of biofilm whilst generating electricity.

This study aims to evaluate the fate of toxic materials that may enter MFC cascades on power generation and the response of the microbial community to these bactericidal agents (ampicillin and chloroxylenol). These are examples of materials that an MFC unit set up in the field/homes could be exposed. Sequencing analysis of the microbial ecology was also carried out before and after the introduction of these materials to evaluate their impact on the microbial community. To the best author’s knowledge, this is the first time that the effect of these materials on urine fuelled MFCs is reported.

## 2 Materials and methods

### 2.1 Reactor configuration

MFCs were assembled using terracotta ceramic cylinders sealed at one end (Orwell Aquatics, UK) with the following dimensions: length 10 cm, outside diameter 2.9 cm, inside diameter 2.1 cm, wall thickness 4 mm. The anode electrode was made of carbon veil (carbon loading 20 mg/cm^2^) with a macro surface area of 300 cm^2^, which was folded and wrapped around the terracotta cylinder with the use of nickel chromium (Ni-Cr) wire for current collection. The cathode was made of activated carbon (30% wet proofed with polytetrafluoroethylene) as previously described [17]. The 30 cm^2^ activated carbon-coated cathode was inserted into the cylinder and connected via stainless steel crocodile clip. The MFC was placed in a plastic container (~30 mL working volume) where the outer anode surface was fully immersed into the anolyte (urine).

### 2.2 MFC operation

The set-up was a cascade of six MFCs operated under an external loading (MFC 1–6). The MFCs within each cascade were fed from the same feedstock line, but with air gaps, which allowed isolation between units. The MFC cascade was inoculated with activated sewage sludge (Wessex Water Scientific Laboratory, Saltford, UK) in batch mode during the first 2 days (day 1; 100% sludge, day 2; 50:50 v/v sludge: urine) and then fed in continuous mode with urine from day 3. The anodic biofilm was established over a 90-day period with steady power generation prior to the testing. The feedstock consisted of human urine at pH of ~ 7.2 in a flow rate of 400 mL/day and a hydraulic retention time of approximately 3.5 h. The external loading applied to the closed circuit MFC cascade was 100 Ω during the experiment. The antimicrobial agents were mixed with the outlet urine (5 g/L of ampicillin (x1 is 50 mg/L) and 4.8 g/L (x1 is 48 mg/L)) of chloroxylenol, according to manufacturer’s instruction. Table 1 shows the characteristics of the two bactericidal agents.

**Table 1:** Physicochemical properties of chemical agents applied in this study.

	Ampicillin	Chloroxylenol
Common use	Antibiotics	Antiseptic, disinfectant
Molecular structure		
Molecular formula	C_16_H_19_N_3_O_4_S	C_8_H_9_OCl
Mol. weight (g/mol)	349.4	156.61
Melting point (°C)	208	115
pKa	2.5 (7.3 at 25 °C)	9.76

Source: https://pubchem.ncbi.nlm.nih.gov/compound.

### 2.3 Ampicillin analysis

Chromatographic separation and mass spectrometric detection of ampicillin was carried out using Waters Acquity UPLC (Ultra High Performance Liquid Chromatography) system equipped with Waters TQ Detector (Waters, Milford, USA). The UPLC system consists of a binary solvent manager, a sample manager and a column thermostat. Electrospray ionization (ESI)-MS detection was carried out in positive ion detection mode. The UPLC-MS/MS instrument was controlled by Waters MassLynx software version 4.1 (Waters, Milford, USA). Data analysis was carried out using TargetLynx software version 4.1 (Waters, Milford, USA). For the chromatographic separation of ampicillin, 0.1% formic acid in water (mobile phase A) and 0.1% formic acid in methanol (mobile phase B) with gradient elution and a reversed phase analytical column (50 mm × 2.1 mm; 1.7 µm Acquity UPLC BEH C18) was used. Separation was obtained using the gradient program starting from 5% of mobile phase B for the first 1.2 min (directed to the waste), mobile phase B content was raised to 100% over 2.3 min and kept at 100% for 1 min, thereafter lowered again to 5% over 0.2 min and kept at 5% of mobile phase B for 1.8 min. Eluent flow rate was 0.25 mL/min. Electrospray (ESI) interface was in use for the mass-spectrometric detection in the positive multiple reaction monitoring (MRM) mode for detection of ampicillin. Triple quadrupole detector transitions *m*/*z* 350 [M+H]^+^ -> *m*/*z* 160; 106 (for ampicillin); and *m*/*z* 355 [M+H]^+^ -> *m*/*z* 160; 111 (for ampicillin-D_5_, IS) were used for quantification and qualification. Optimised parameters for ESI and MS were used with capillary voltage of 0.8 kV, cone voltage 31 V, source temperature 120 °C, desolvation gas temperature 350 °C and desolvation gas flow rate 800 L/h and cone gas flow rate 30 L/h. All samples were analysed in duplicates. Back calculated concentrations using linear regression fitting with 1/x^2^ weighting was used to quantify samples.

### 2.4 Microbial analysis

After 90 days of operation and with stable power generation, the anodic biofilm bacteria were collected 24 h before and 24 h after the chemical treatments and analysed. Metagenomic DNA from samples were extracted in duplicate using Dneasy Blood & Tissue kit (Qiagen, Germantown, MD) according to kit protocol. DNA quality and quantity were measured by Nano-Drop Lite spectrophotometer (Thermo Fisher Scientific, Waltham, MA, U.S.A.). Bacterial 16S rRNA gene was amplified using universal primers 1055f (ATGGCTGTCGTCAGCT) and 1392r (ACGGGCGGTGTGTAC) [18] and barcoded fusion primers with sequencing adaptors [19]. 16S amplicon sequence‘s quality and quantity of was checked with bioanalyser (Agilent Technologies 2100, CA, U.S.A.). 16S amplicon sequencing was performed using an Ion Torrent PGM (Thermo Fisher, MA, U.S.A.) platform with Ion Torrent 318v2 Ion Chip by following manufacturer’s instructions (Ion PGM Hi-Q Sequencing kit, Product no. MAN0009816). All 16S rRNA gene raw sequences have been deposited to Sequence Read Archive (SRA) at The National Center for Biotechnology Information (NCBI) under the accession no. SAMN12583022-SAMN12583029. Qiime2 V.2018.11 [20] pipeline was used 16S amplicon data analysis. Briefly, quality check and chimera removal of 16S amplicon reads were performed dada2 command. Taxonomic classification was performed by consensus-blast command against Silva ribosomal databases v132 [21].

## 3 Results

### 3.1 MFC power performance

**Fig. 1:**
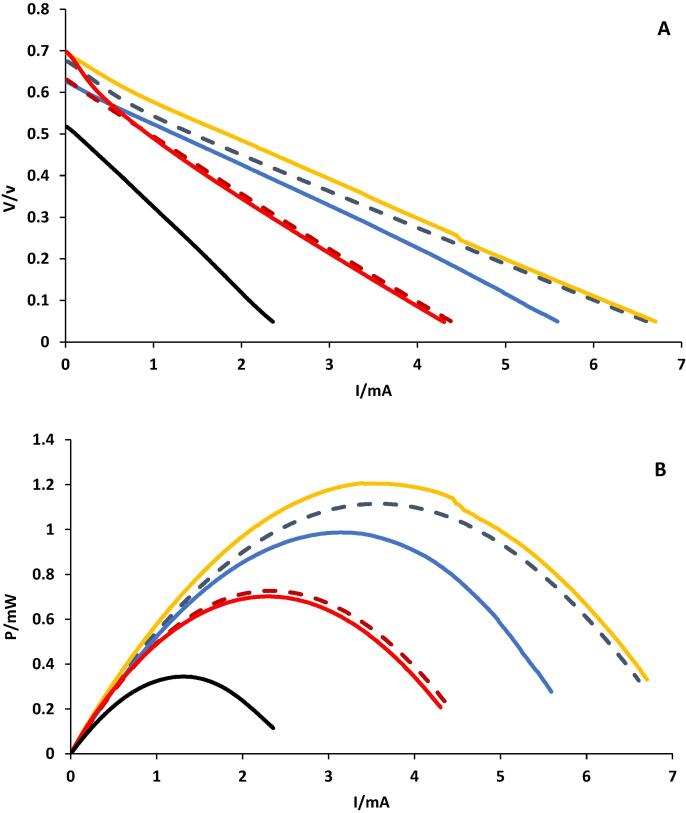
Polarisation (A) and power curves (B) from cascade of six urine fed-MFCs prior to the addition of bactericial agents. Number represent MFC positions within the cascade. Dash Blue-gray line MFC 1., Orange line MFC 2, Blue line MFC3., Dash dark red line, MFC4., Red line, MFC5., Black line MFC 6. (For interpretation of the references to colour in this figure legend, the reader is referred to the web version of this article.)

Fig. 1A and B show the polarisation and power curves respectively, obtained from the polarisation experiments of the individual MFCs within the cascade. They show a correlation between the MFC position in the cascade and the power generation, thought to be due to the differences in the substrate composition as a result of nutrient depletion.

For instance, MFC 2 produced almost two times more power (1.2mW) than MFC 4 (0.7mW), which in turn produced two times more power than MFC 6 (0.35mW), which received a more depleted urine feed. These results are similar to power outputs from similar MFCs previously reported [10].

**Fig. 2:**
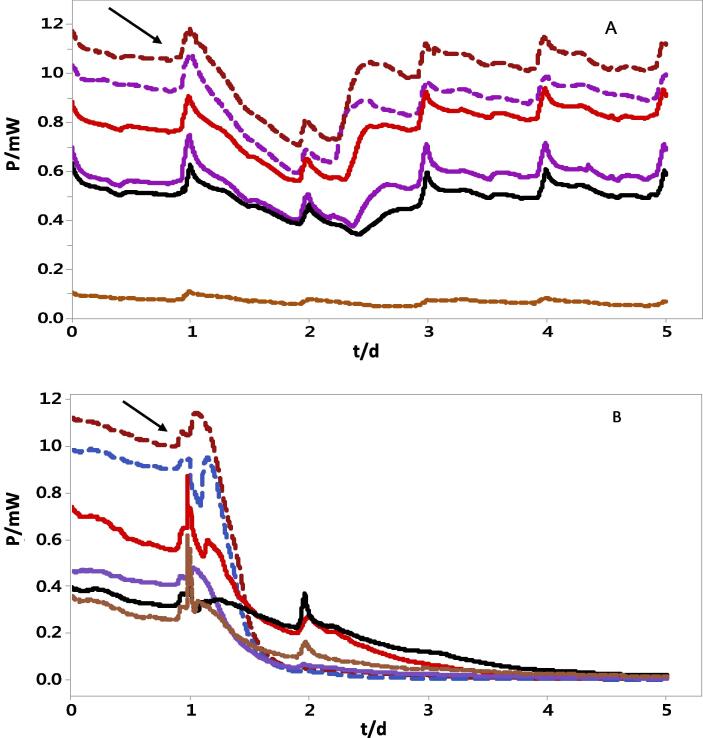
(A) Power generation during the addition of ampicillin (5 g/L) to the closed circuit cascade (B) Power generation during the addition of chloroxylenol (4.8 g/L) to the closed circuit cascade. Number represent individual MFC position within the cascade. Dash Blue line MFC 1., Dash dark-red line MFC 2., Red line MFC3., Blue line MFC4., Black line MFC5., Red square-dot line MFC 6. (For interpretation of the references to colour in this figure legend, the reader is referred to the web version of this article.)

Fig. 2A shows power generation before, during and after the addtion of ampicillin to the cascade. Prior to the addition of the antibiotics, power generation was stable around

1.2 mW, however, the introduction of ampicillin brought about varying levels of impact on the MFCs performance. For instance, the power output by MFC 2 decreased from 1.2 mW to 0.8 mW within 24 h of ampicillin addition compared to a reduction from 0.6mW to 0.5 mW in MFC 5. This is an indication of a gradual decline in the overall concentration of the antibiotics, which could be attributed to various phenomena such as adsorption, biosorption and general degradation along the cascade. Nevertheless, the resullts showed a complete recovery from the effects of the antibiotics within 48 h, with power generation returning to pre-antibiotic introduction stages across all MFCs (Fig. 2A).

On the other hand, Fig. 2B shows the strong impact of chloroxylenol on the biofilm community. Upon the addition of the bactericidal agent, there was an immediate and significant decline in power generation in all MFCs within the cascade. Although the bactericidal agent was applied during a 12 h period, the detrimental effect lasted more than 4 days afterwards with no sign of recovery. Power generation went to near zero in all the MFCs by day 5 of the experiment, an indication of the destruction, rather than inhibition of the electroactive bacteria or their metaboilic activities. Evaluation of the MFC anodes indicated that chloroxylenol was absorbed within the anode and remained potent for a long period of time with noticeable strong odour several days after the test. This shows that chloroxylenol was stable and potent throughout the test period, thereby able to casue significant damage to the microbial community. The destruction of the bacterial community by chloroxylenol could be due to the high dosage utilised (4.8 g/L), which was more than the normal recommended dosage (48 mg/L). The biofilm might have been able to degrade and recover from the impact of a lower dosage as lower concentrations favours degradation via co-metabolism [22]. Most of the phamaceutical degradation and removals via BES systems reported in literature are in the ng-µg/L concentration

### 3.2 Microbial community analysis

#### 3.2.1 Impact of ampicillin on the bacterial community

**Fig. 3:**
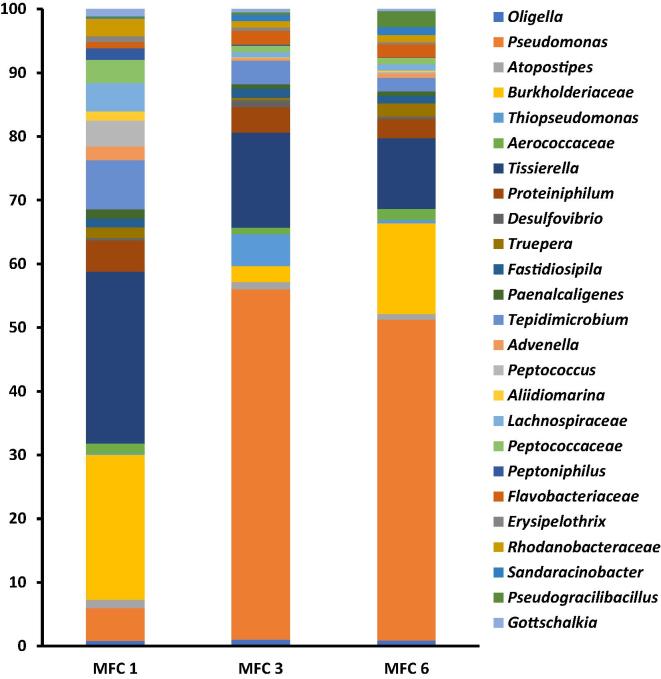
Genera distribution of bacterial community of the developed anode of urine-fed MFC 1, 3 and 6 prior to the addition of ampicillin. Only strains up to 1% shown.

To understand the effects of the bactericidal agents on the microbial community, anodic microbal community analysis was carried out before and after the treatment. The results showed a substatially diverse community with at least 36 genera detected within the cascade. The genus *Pseudomonas* was the dominant community within MFC 3 and 6 consitituting 55% and 50% of the community respectively. MFC 1 on the other hand was dominated by bacteria related to the genus *Tissierella* (27%) and *Burkholderiaceae* (22%) (Fig. 3).

*Psuedomonas* sp are important electroactive bacteria and carry out significantly greater electron transfer in the presence of an electrode. Earlier reports suggest that members of the genus *Pseudomonas* produce compounds such as phenazine pyocyanin, which function as electron shuttles between the bacteria and an electron acceptor such as electrodes. Their abundance in any MFC system perhaps is an indication of efficient electron transfer, leading to improved power generation [23].

The abundance of bacteria related to the genus *Tissierella* in MFC 1, which received neat untreated urine, shows their importance to urine metabolism, ammonia production and overall power generation processes. Previous reports have shown that members of *Tissierella* utilise creatinine, an important component of urine as their sole carbon source. In most *Tissierella*, creatinine metabolism occurs via N-methylhydantoin, N-carbamoyl-sarcosine, and sarcosine to acetate, ammonia, and CO_2_. Creatinine can also be degraded by members of *Tissierella,* via creatine, sarcosine and glycine to products such as acetate, mono-methylamine, ammonia and carbon dioxide [24], [25]. The family *Burkholderiaceae,* which was also dominant in all MFCs, contain many known electroactive bacteria [26] and could be responsible for power generation in the MFC cascade under investigation. Examples of known electroactive bacteria within *Burkholderiaceae* include *Rhodoferax sp* and *Cupriavidus sp*., both of which carry out extracellular electron transfers through direct and mediated electron transfers to electrode surfaces [27]. The presence of different genera of bacteria within the anodic biofilm demonstrates the importance of microbial cooperation and interactions for substrate degradation and concomitant electricity generation with individual MFC. The substrate (neat human urine) utilised in this study might also have contributed to the selection of the type of bacterial community present on the anode. Research has shown that the type of substrate fed into a MFC was a strong determinant of the composition of the microbial community as well as the MFC performance including power density [27].

**Fig. 4:**
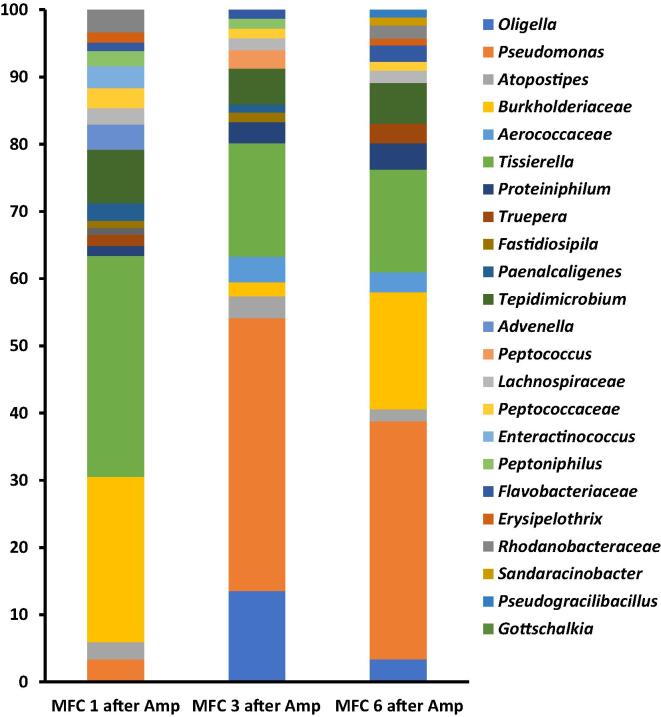
Genera distribution of bacterial community of the developed anode of urine-fed MFC 1, 3 and 6 24 h after the addition of ampicillin. Only strains up to 1% shown.

Analysis of the bacterial community after the addition of ampicillin showed significant changes within the community. For instance, there was a general decline of 2%, 17% and 16% in the proportion of *Psuedomonas* in MFC 1, 3 and 6 respectively (Fig. 4). This decline perhaps was responsible for the temporary drop in power generation within the 12 h of ampicillin addition, since other community remained largely stable, except for members of *Oligella* which increased slightly in MFC 3 and 6 (Fig. 4). Apart from the decline in the proportions of some of the dominant strains, some bacterial strains were lost after ampicillin addition. Examples include *Oligella Peptococcus* and *Gottschalkia* which were lost in MFC 1 and *Truepera*, *Advenella*, *Erysipelothrix* lost in MFC 3, while *Fastidiosipila Advenella* were lost in MFC 6. Previous research involving the use of ampicillin in microbial fuel cell systems showed that single culture anodic bacterial was susceptible to ampicillin addition. For instance, the addition of 40 mg/L of ampicillin to a microbial fuel cell based sensor platform resulted in no metabolic activity or electrical signal from *E. coli* or *Staphylococcus aureus* inoculated systems [28]. Meanwhile the addition of 200 mg/L of ampicillin to an *E coli* based biofilm on nanopillars resulted in considerable voltage decline [29]. These examples highlight the robustness and stability of mixed-culture anodic biofilm compared to mono-culture biofilms.

Furthermore, the abilities of anodic biofilm to withstand high concentration of antibiotics have been linked to the secretion of extracellular polymeric substances (EPS) by microorganisms. EPS is made up of complex materials containing proteins, polysaccharides, nucleic acids, humic acids and other organic macromolecules. They are made up of several functional groups which interfere with toxic materials and provide protection for the microbial community [30], [31]). Research has shown that the secretion of EPS is enhanced in the presence of antibiotics and other inhibitory materials and that biofilms without EPS are particularly more vulnerable to antibiotics as they lack the increased protection provided by EPS. EPS has also been demonstrated as a reservoir for the containment and removal of toxic substances from the biofilm suspension, thus shielding the microbial community from their harmful effects [32], [33]. The presence of EPS was visible within the MFCs in the current study and it could have potentially enhanced the resilience of the microbial community during the introduction of the chemicals.

#### 3.2.2 Impact of chloroxylenol on the bacterial community

Prior to the addition of the chloroxylenol to the cascade, there was a stable power generation from all MFCs within the cascade (Fig. 2B). Addition of choloxylenol resulted in an immediate loss of power generation over a period of 4 days. To evaluate the response of the bacterial community to the introduction of chloroxylenol, the biofilm from the anode was analysed before and after the introduction of chloroxylenol.

**Fig. 5:**
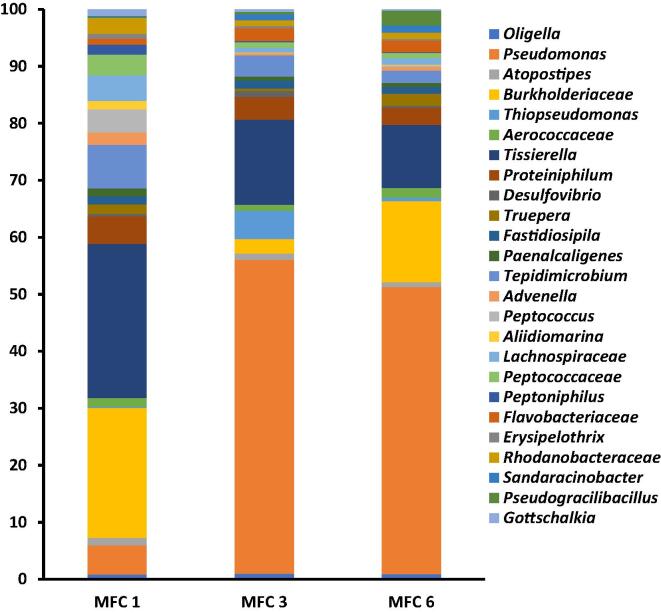
Genera distribution of bacterial community of the developed anode of urine-fed MFC 1, 3 and 6 prior to the addition of chloroxylenol. Only strains up to 1% shown. Fig. 5 shows the results of the analsysis of the bacteria community prior to the addition.

The results showed at least 25 genera (those > 1% of the community) were detected prior to the addition of chloroxylenol. Members of the genera *Tissierella* and *Burkholderiaceae* were the dominant strains within MFC1 while bacteria related to the genus *Pseudomonas* were the dominant community within MFC 3 and 6, constituting 55% and 50% respectively (Fig. 5).

**Fig. 6:**
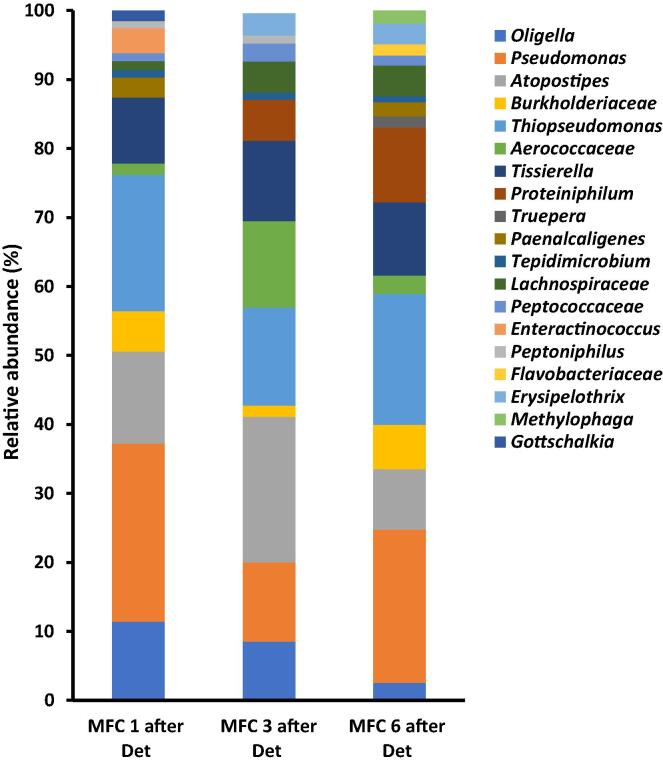
Genera distribution of bacterial community of the developed anode of urine-fed MFC 1, 3 and 6 24 h after the addition of chloroxylenol. Only strains up to 1% shown.

Analysis of the bacterial community after the addition of chloroxylenol showed a significant shift in the community (Fig. 6). In MFC1, for instance, the proportion of dominant *Tissierella* and *Burkholderiaceae* declined by 18% each respectively, while the proportion of *Pseudomonas, Thiopseudomonas, Atopostipes* and *Oligella* increased by 20%, 19%, 12% and 10% respectively. The decline in the proportion of members known for urine metabolism as well as those with electroactive abilities could explain the drastic decline and loss of power generation after the treatment. Moreover, recorded increases in the proportion of some strains might not be as a result of general increase in their number, but perhaps due to the loss of some strains and the greater decline recorded in the other dominant strains. Previous research has shown that for sustained power generation in MFC systems, the number of bacteria within the anodic chamber is critical. A report suggest that the minimum number of bacteria needed to sustain proper functioning of the MFC system as 10^6^ CFU/ml [28]. As such, there was the possibility of the decline in anodic bacterial community below this threshold after the introduction of chloroxylenol, resulting in the significant loss of power generation recorded (Fig. 2B). Introduction of chloroxylenol into the MFC cascade also resulted in the loss of at least 6 genera (for those > 1%) and 13 genera in total. In MFC 1 for instance, the genera *Proteiniphilum, Truepera, Flavobacteriaceae, Erysipelothrix and Methylophaga* were lost*.* While *Paenalcaligenes*, *Truepera*, *Enteractinococcus*, *Methylophaga* and *Gottschalkia* were lost in MFC 3 and, *Enteractinococcus*, *Peptoniphilus* and *Gottschalkia* were lost in MFC 6. Generally, the results show that chloroxylenol (being a disinfectant) was a more potent bactericidal agent than ampicillin (an antibiotic), partly due to its impacts on a wider range of microorganisms. It might also be due to its better stability within the anode chamber thereby maintaining its efficacy over a longer period, unlike ampicillin.

One pertinent observation from this study is the total loss of power generation during the SRX^0^??responsible for power generation were significantly inhibited or killed. Specifically, this observation suggests the destruction of bacterial species that are important for urine conversion or those responsible for electron transfer. For instance, the genera *Gottschalkia* which was lost in most of the MFCs after the addition of chloroxylenol are known for the utilisation of uric acid as the sole carbon and energy source, resulting in the generation of acetate, formate, CO_2_ and ammonia [34]. The destruction of *Gottschalkia* with the introduction of chloroxylenol within the cascade could have resulted in the poor utilisation of a major component of the urine feedstock (uric acid) and the lack of suitable substrates such as acetate for electrogenic communities.

**Fig. 7:**
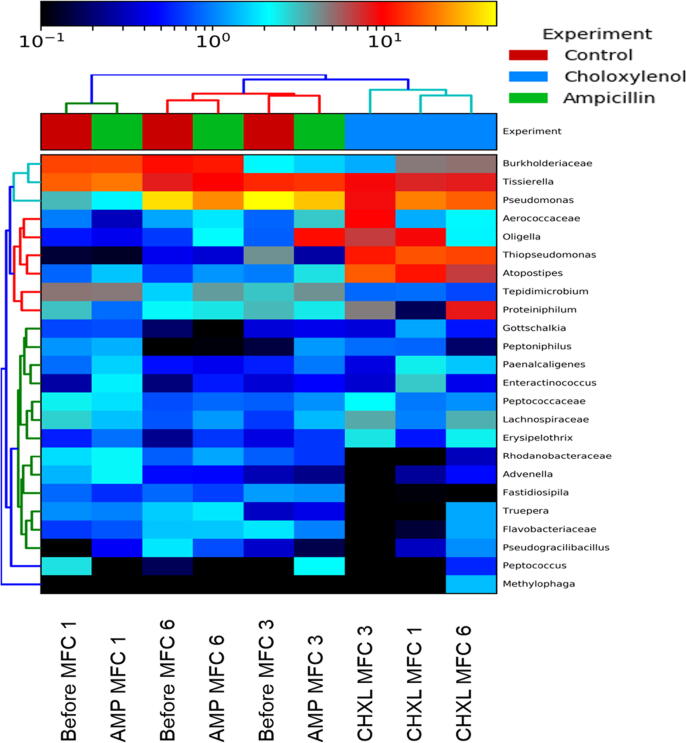
Heat map representation and cluster analysis of the impacts of the addition of bactericidal agents to the MFC cascade. The horizontal clustering indicates the similarity of species while vertical clustering shows the different treatments. The colours show the significance of the impact, with black representing the negative of log10 of the loss of species, and yellow/red representing the positive log10 of species richness/abundance. Control: MFC community prior to the introduction of the chemical agents. AMP: after ampicillin test, CHXL: after chloroxylenol test. (For interpretation of the references to colour in this figure legend, the reader is referred to the web version of this article.)

*Proteiniphilum* which was also lost within the cascade has been previously reported as having the ability to metabolise a wide range of polysaccharides including cellulose, hemicellulose, chitin, starch and fructan. It also encodes extracellular and intracellular proteases involved in protein and oligopeptide degradation. The end products of its metabolic activities include carbon dioxide, hydrogen, acetate, formate, propionate which are suitable substrates for electrogenesis [35]. Fig. 7 showed the community distribution before and after the addition of the biocides. It summarises and highlights the impacts of the chemical agents on the microbial community.

#### 3.2.3 Degradation of ampicillin within the microbial fuel cell cascade

The introduction of ampicillin into the MFC cascade in the present study, resulted in only a temporary decline in power generation. This was followed by immediate recovery of power generation, an indication that the electroactive community were able to quickly recover from the impact of ampicillin. To understand the fate of ampicillin within the cascade, anolyte samples were collected 24 h before and after ampicillin addition and analysed.

**Fig. 8:**
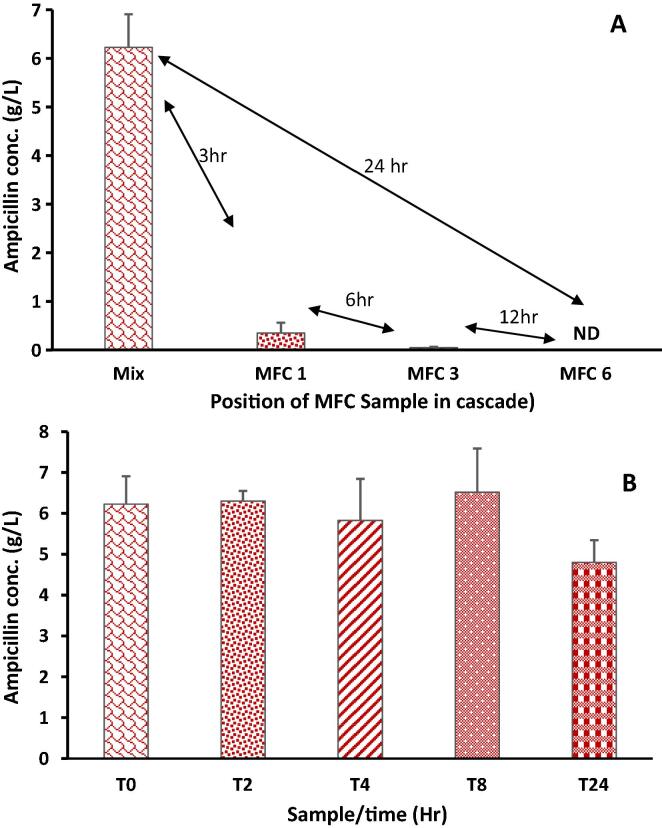
Degradation of ampicillin within (A) a cascade of 6 MFCs and (B) within urine outside of microbial fuel cells. Mix = 5.5g/L ampicillin in urine, ND: not detected.

The result showed a significant degradation of the ampicillin within the cascade beginning from MFC 1, with retention time of only 3 h resulting in about 95% decline in the concentration of ampicillin, and 99% after the treatment in MFC3 (Fig. 8A). At the end of the treatment within the entire cascade (MFC6), the concentration of ampicillin was below detection, an indication of complete breakdown of the antibiotics within 24 h. The rapid degradation of ampicillin within the MFCs helped the microbial community avoid the harmful impacts of the high concentration of the antibiotics. In contrast, in the control samples (containing same concentration of antibiotics in urine, but outside of an MFC) only about 17% degradation of the antibiotics was recorded after 24 h (Fig. 8B). This is an indication that the conditions within the anodic chamber of the active MFCs aided the degradation of the antibiotics. A previous research which evaluated electrochemical oxidation of up to 1 g/L of ampicillin in a boron-doped diamond electrode in batch reactor recorded 97.1% degradation of the ampicillin [36]. The treatment 50 mg/L of penicillin (an antibiotic similar to ampicillin) in a single chamber MFC was reported with 98% removal efficacy [22]. Furthermore, a review of the degradation of some pharmaceuticals within bio-electrochemical systems showed that the introduction of 500 ng/L of norfluoxetine and 500 ng/L of primidone into dual chamber MFC resulted in the removal efficiency of 98% and 21% respectively [22]. This is an indication of the differential responses of bio-electrochemical systems to different chemical agents. The concentration of chemical biocides applied in the current study is significantly higher than those reported in literature.

Previous research has shown that bioelectrochemical systems such as microbial fuel cells are capable of degrading different pharmaceuticals. Amongst these are ampicillin [11], sulphonamides [37], Florfenicol and Ofloxacin [38]. Reports by Zhou et al. [39] on the electrochemical responses of antibiotics addition to MFCs with developed anode showed that lower concentration of antibiotics resulted in greater power generation, whilst higher concentrations generated at slightly lower voltage levels. The research also highlighted the possibility of an enhanced NH_3_-N removal in the presence of antibiotics resulting in increased efficiency from 57% to 76%. The research showed that when up to 31 µg/L of aureomycin, 5.3 µg/L of roxithromycin and 5.2 µg/L of norfloxacin were added to MFC fed with simulated animal wastewater, the concentrations of the antibiotics were below detection in the effluent, an indication of complete degradation within the MFCs.

The differences recorded in the response of the microbial community in the current study to the introduction of ampicillin and chloroxylenol, highlights a unique form of selective response by the anodic biofilm. Essentially, whilst ampicillin was effectively removed from the anodic solution, chloroxylenol remained largely unaffected over several days resulting in significant inhibition of power generation. This tendency of anodic biofilm to response differentially to different chemical agents has been previously reported. For instance, Harnisch at al. [37] reported the complete removal of sulfamethoxazole by the electroactive biofilms of MFCs fed with artificial wastewater while the concentration of sulfathiazole remained largely unchanged. In a similar manner, sulfadiazine was partly removed by the MFCs while sulfadimidine was unaffected. These results suggest the substance-specific bio-transformational tendencies of microbial biofilms, a tendency which is perhaps dependent on the nature and composition of the microbial community. There are a few limitations with the current study in terms of scope and depth. Firstly, only 2 biocides were tested, even though there are others that could be exposed to working MFCs. As such, we only chose an example for antibiotics and another for antiseptic biocides. The concentrations of chloroxylenol could not be measured due to lack of facility. EPS concentration was not also determined but were observed to be present in the MFCs. Further investigation will be required to evaluate these and the degradation pathways and possible metabolism of these chemical agents.

## 4 Conclusion

This study assesses the impact of two common bactericidal chemical agents on power generation as well as the response of the microbial community to these chemical agents within MFC cascades. The results show that chemical agents, depending on their composition and concentration affect the microbial community and power generation in microbial fuel cell differently. It highlights the unique substance-selective tendencies of MFC biofilm during exposure to different chemical agents. This knowledge could prove valuable for future deployment of MFC technology to location further afield, where potential contaminants or other inhibitory materials could be introduced into the MFC systems. Moreover, further research would be required to reveal the underlying mechanisms of microbial degradation of bactericidal agents/pollutants, the metabolic pathways involved as well as the by-products of those bio-transformation reactions.

## Declaration of Competing Interest

The authors declare that they have no known competing financial interests or personal relationships that could have appeared to influence the work reported in this paper.
